# Asymptomatic urinary tract infections and associated risk factors in Pakistani Muslim type 2 diabetic patients

**DOI:** 10.1186/s12879-021-06106-7

**Published:** 2021-04-26

**Authors:** Azizul Hasan Aamir, Umar Yousuf Raja, Ali Asghar, Saeed Ahmed Mahar, Tahir Ghaffar, Ibrar Ahmed, Faisal Masood Qureshi, Jamal Zafar, Mohammad Imtiaz Hasan, Amna Riaz, Syed Abbas Raza, Irshad Ahmed Khosa, Jahanzeb Khan, Mahwish Raza, Jaffer Bin Baqar

**Affiliations:** 1 Khyber Girls Medical College, Hayatabad Medical complex, Peshawar, Pakistan; 2 Post Graduate Medical Institute, Peshawar, Pakistan; 3 Shifa International Hospital, Islamabad, Pakistan; 4 Fatimiyah Hospital, Karachi, Pakistan; 5 National Institute of Cardiovascular Diseases, Karachi, Pakistan; 6 Lady Reading Hospital, Peshawar, Pakistan; 7 Al-Khaliq Hospital, Multan, Pakistan; 8 Hanif Medical Center, Rawalpinidi, Pakistan; 9 Diabetes Institute of Pakistan, Lahore, Pakistan; 10 Jinnah Hospital, Lahore, Pakistan; 11 National Defence Center, Lahore, Pakistan; 12 Balochistan Medical Center, Quetta, Pakistan; 13 Dow University of Health Sciences, Karachi, Pakistan; 14 Shaheed Zulfikar Ali Bhutto Institute of Science and Technology, Karachi, Pakistan; 15 University of Karachi, Karachi, Pakistan

**Keywords:** Type II diabetes mellitus, Urinary tract infections, Asymptomatic, Pakistani Muslim population

## Abstract

**Background:**

One of the leading long-term complications of type 2 diabetes mellitus (T2DM) includes renal dysfunction and urinary tract infections (UTI) which are considered to be prevalent in uncontrolled diabetes. Moreover, physiological factors like age, gender, duration of diabetes, other diabetic complications like neuropathy, autonomic neuropathy and glycosuria are also considered as predisposing factors for increased prevalence of UTI in diabetes which can be symptomatic or asymptomatic.

**Methods:**

This was a cross-sectional, multi-centre study including diabetic patients from 12 clinical sites spread across major cities of Pakistan. The inclusion criteria were adult Pakistani population of age between 18 to 75 years both genders and suffering from T2DM irrespective of duration. A detailed clinical history of the past 3 months was recorded and, biochemical investigations of blood samples were conducted. Urine culture analysis performed identified the type of pathogen present and was done only for asymptomatic patients.

**Results:**

A total of 745 type 2 diabetic patients were initially screened, out of 545 patients considered for final analysis 501 (91.92%) were negative and the rest 44 (8.08%) had positive urine culture. Female gender had a significantly higher proportion of positive urine culture (77.27%, *p*-value< 0.001). Body mass index and mean age had insignificant distribution among the two groups of positive and negative urine culture, with age 40–59 years having higher proportion (70.45%) in the positive group. *Escherichia coli* was detected in most of the positive samples (52.3%). All bacterial samples were found resistant to Ciprofloxacin.

**Conclusion:**

Diabetic Pakistani muslim female patients are identified to be at high risk of suffering from asymptomatic UTI and age more than 40 years is an important risk factor. *Escherichia coli* was the most common causative organism among people living in this geographical area.

## Background

Type 2 Diabetes mellitus (T2DM) has become a global disease with millions suffering [1], primarily due to onset at a relatively early age because of a sedentary lifestyle and other contributing factors [2]. Pakistan is also under the huge prevailing burden of T2DM, and health statistics estimates report 16.98% of the Pakistani population is currently living with T2DM [3]. Moreover, uncontrolled, or untreated hyperglycaemia leads to micro and macro-vascular complications, which itself pose a considerable risk of premature death in T2DM patients [4]. Accompanying financial burden and access to care adds further complexity in the treatment of T2DM patients with multiple comorbidities [5].

One of the leading long-term complications of T2DM includes renal dysfunction and associated urinary tract infections (UTI). High glucose concentration in urine promotes urinary colonization of microorganisms, and the patient becomes more prone to microvascular disease of the kidneys. This has also become a major concern as many studies have reported a high prevalence of UTI in T2DM patients [6]. Clinical profile of patients with diabetes shows poor circulation, decreased immune system due to reduced ability of white blood cells to fight infections, poor contractions of the bladder leading to bladder dysfunction are some of the contributing factors leading to increased cases of UTI among diabetics [7]. Moreover, physiological factors like age, gender, duration of diabetes, long term use of anti-diabetic drugs, other diabetic complications like neuropathy, glycosuria are also considered as predisposing factors for increased prevalence of UTI in diabetics. UTI can be symptomatic or asymptomatic in patients with diabetes and encompasses asymptomatic bacteriuria (ABU), urethritis, cystitis, prostatitis and pyelonephritis [8].

Hence, this study was conducted to screen T2DM patients to determine the frequency of asymptomatic state of UTI based on clinical, pathological, medical and treatment history and other contributing risk factors in Pakistani Muslim population.

## Methods

It was a cross-sectional, multi-centre study including diabetic patients presenting at 12 clinical sites spread across major cities of Pakistan, including Karachi (*n* = 2), Lahore (*n* = 3), Islamabad (*n* = 2), Peshawar (*n* = 3), Multan (*n* = 1) and Quetta (*n* = 1). The study was conducted simultaneously at all sites for the period between June-2019 to May-2020.

The study included adult Pakistani Muslim population of age between 18 to 75 years from both genders, volunteering to participate by giving written informed consent, and suffering from T2DM irrespective of duration. Through consecutive (non-probability sampling technique) patients were screened at all sites for recruitment.

After explaining the study procedures and consent process, data obtained from participants regarding demographics including age, gender, and body mass index. A detailed clinical history of past 3 months was acquired regarding the diagnosis of urinary tract infection based on commonly reported symptoms like frequent urination, urgency, burning micturition, fungal infections, benign prostate hyperplasia (male patients only), premenopausal symptoms (female patients only) and were excluded from the study. Along with urine culture biochemical investigations of blood samples were conducted at designated laboratories for the estimation of glycosylated haemoglobin (HbA1c) levels, serum ketones, liver function tests including Alanine aminotransferase (ALT), Alkaline phosphatase (ALP), Aspartate aminotransferase (AST) and estimated glomerular filtration rate (eGFR). Midstream urine samples were collected under sterile condition within the laboratory for confirmation of diagnosis. Laboratory testing protocols ensured that all urine samples collected for bacterial culture were not contaminated and standard storage conditions were maintained. Urine culture analysis identified the type of pathogen present and was performed only for asymptomatic patients (patients with no history of UTI in the past 3 months). Considering the study limitations, patients with a positive history of UTI based on symptoms were considered symptomatic and they were not referred for a confirmatory test to the laboratory.

Data was entered and analyzed using STATA version 15.0. Baseline characteristics, and laboratory results were compared between patients with the positive and negative outcome of urine culture. The normality of continuous variables was assessed using Shapiro Wilk tests and mean with standard deviation and median with interquartile range was reported according to the distribution. Student’s t-test or Mann Whitney U test was used to assess the significant difference. Frequency and percentages were reported for categorical variables using Chi-square or Fisher’s exact test depending upon cell count assumption for culture-positive and negative groups. Odds Ratio was calculated for age and gender. A *p*-value of < 0.05 was considered as a cut off for a significance for all results.

## Results

**Fig. 1:**
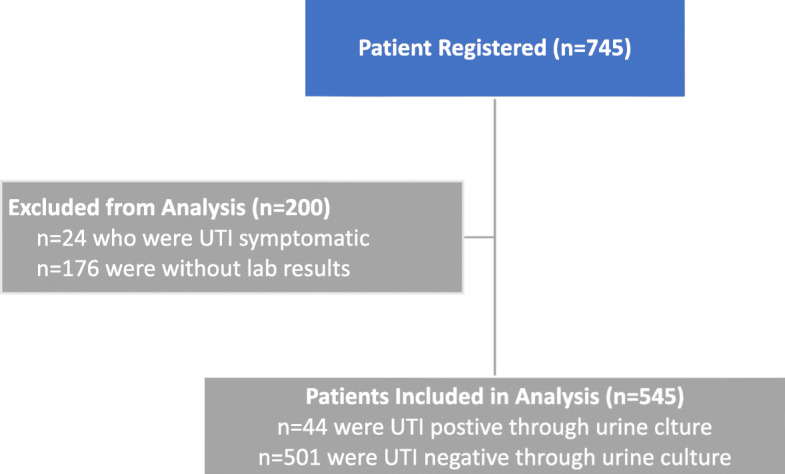
Patient recruitment flow chart

A total of 745 type 2 diabetic patients were initially screened of which 200 (26.8%) participants were considered ineligible for further analysis due to patients not undergoing desired laboratory investigations 176 (23.6%) and symptomatic UTI 24 (3.2%). The final analysis was performed on 545 patients out of which 501 (91.9%) had negative and the rest 44 (8.1%) had positive urine culture. (Fig. 1).

Female gender had a significantly higher proportion of positive urine culture (77.27%, *p*-value< 0.001). Body mass index and mean age had insignificant distribution among the two groups of positive and negative urine culture, with age 40–59 years having higher proportion (70.45%) in the positive group. Table 1 showed the comparison of baseline characteristics among Urine culture positive and negative patients.

**Table 1:** Comparison of study characteristics between UTI and Non UTI patients (*n* = 545)

Study Characteristics	Urine Culture		Total (*n* = 545)	*P*-value
	Negative (***n*** = 501)	Positive (***n*** = 44)		
Gender				
Female	227 (45.3)	34 (77.3)	261 (47.9)	< 0.001*
Male	274 (54.7)	10 (22.7)	284 (52.1)	
Age (years); mean ± SD	49.60 ± 10.8	51.4 ± 8.3	49.8 ± 10.7	0.28
Age Groups, years				
< 40	92 (18.4)	2 (4.5)	94 (17.2)	0.02*
40 or above	409 (81.6)	42 (95.5)	451 (82.8)	
BMI (kg/m^2^); mean ± SD	29.1 ± 4.9	30.0 ± 4.3	29.1 ± 4.8	0.24
BMI Groups				
< 25	95 (19.0)	7 (15.9)	102 (18.7)	0.62
25 or above	406 (81.0)	37 (84.1)	443 (81.3)	

*BMI* Body Mass Index

*significant at *p* < 0.05

**Fig. 2:**
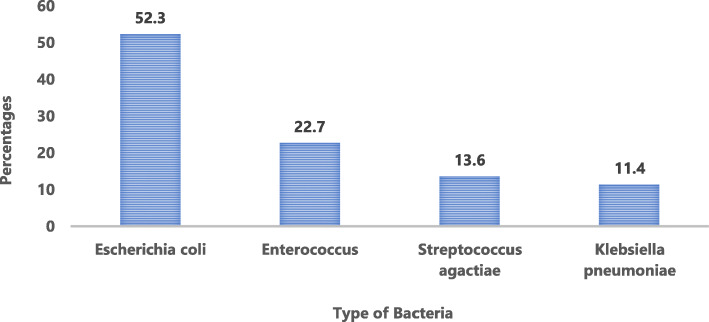
Bacterial pathogens in diabetic patients with positive urine culture (*n* = 44)

*Escherichia coli* (*E. coli*) was detected in most of the positive samples (52.3%), followed by Enterococcus (22.7%), Streptococcus agactaiae (13.6%), and *Klebsiella pneumoniae* (11.4%) (Fig. 2).

Overall, laboratory results of HbA1C, APT, eGFR and serum ketone levels had insignificant distribution between two groups. Patients with HbA1C levels ≥7 had a higher prevalence in the group with positive urine culture (86.4%) (Table 2). Females were 1.7 times more likely to develop Urinary tract infection than males whereas age ≥ 40 years were 1.17 times more likely to acquire UTI (Table 3).

**Table 2:** Biochemical profile of participants (*n* = 545)

Variable	Urine Culture		Total (*n* = 545)	*P*-value
	Negative (*n* = 501)	Positive (*n* = 44)		
HbA1C level; mean ± SD	8.8 ± 1.8	8.5 ± 1.51	8.8 (1.8)	0.31
HbA1C level				
< 7	57 (11.4)	6 (13.6)	63 (11.6)	0.65
≥ 7	444 (88.6)	38 (86.4)	482 (88.4)	
eGFR				
< 45	13 (2.6)	1 (2.3)	14 (2.6)	0.89^^^
45 and above	488 (97.4)	43 (97.7)	531 (97.4)	
eGFR; median (IQR)	97 (35.1)	93.4 (35.8)	97 (34.6)	0.44^~^
ALT level; median (IQR)	32 (29.5)	27 (19.5)	32 (28.5)	0.02^*~^
ALT level, IU/L				
Normal (M < 45, F < 34)	304 (60.4)	32 (72.7)	336 (61.7)	
High (M = 45 & above, F = 34 & above)	197 (39.3)	12 (27.3)	209 (38.3)	0.12
AST level; median (IQR)	28 (15)	23 (11.8)	27 (15)	0.01*~
AST level, IU/L				
Normal (M < 35, F < 31)	339 (67.7)	33 (75.0)	372 (68.3)	0.32
High (M = 35 & above, F = 31 & above)	162 (32.3)	11 (25.0)	173 (31.7)	

significant at *p* < 0.05. ^Fischer exact test was applied. ~Mann Whitney U test applied for non-normal data

ALT Normal level: Male < 45 IU/L and Female < 34 IU/L; ALT High level Male: =45 IU/L & above, Female = 34 IU/L & above; AST Normal level: Male < 35 IU/L and Female < 31 IU/L; AST High level: Male =35 IU/L & above, Female =31 IU/L & above

*ALT* Alanine aminotransferase, *AST* Aspartate aminotransferase, *eGFR* Estimated Glomerular Filtration Rate (mL/min)

**Table 3:** Risk Estimation for predicting positive urine culture

Variables	OR	95% C.I. for OR
Gender		
Male	0.416	0.240–0.721
Female	1.705	1.415–2.056
Age		
< 40 years	0.248	0.063–0.971
≥ 40 years	1.169	1.083–1.262
BMI		
< 25	0.839	0.415–1.695
≥ 25	1.038	0.906–1.188
HbA1c		
< 7	1.199	0.548–2.622
≥ 7	0.975	0.863–1.100

*OR* Odds ratio, *C.I*. Confidence interval, *BMI* Body mass index

Bacterial colonies were then assessed for antibiotic resistance in patients with positive urine culture. E.coli was found to be most resistant bacteria against most of the antibiotics with highest resistance with Nalidixic acid (52.2%), followed by Ciprofloxacin, Ceftriaxone and Cefotaxmine (34.7%). Enterococcus Sp. also showed resistance against Doxycycline (40%) and Ciprofloxacin (30%). All bacterial samples were found resistant to Ciprofloxacin with varying strength of 30 to 60% (Table 4).

**Table 4:** Analysis of antibiotic resistance of pathogens found in diabetic patients with positive urine culture

	E.coli (***n*** = 23)	Enterococcus Sp. (***n*** = 10)	***Klebsiella pneumoniae*** (***n*** = 6)	Streptococcus agactiae (***n*** = 5)
**Amoxicillin clavulanic acid**	26.1%	–	–	–
**Cefuroxime**	4.35%	–	–	–
**Ceftriaxone**	34.7%	–	–	–
**Cefixime**	4.35%	–	–	–
**Cefotaxime**	34.7%	–	–	–
**Amikacin**	4.35%	–	–	–
**Gentamicin**	17.4%	10%	–	20%
**Doxycycline**	21.7%	40%	–	–
**Nalidixic Acid**	52.2%	10%	16.6%	–
**Fosfomycin**	–	10%	–	20%
**Ciprofloxacin**	34.7%	30%	33.3%	60%
**Levofloxacin**	–	10%	–	–
**Trimethoprim-sulphamethaxazole**	21.7%	10%	16.6%	–
**Clindamycin**	–	10%	–	–

## Discussion

We investigated the clinical profile of type 2 diabetes patients through a multisite study to assess the prevalence and associated factors of asymptomatic urinary tract infection (UTI) in adult Pakistani Muslim population. Using bacterial culture as standard diagnostic laboratory testing, a prevalence of 8.1% was observed in the study population with *E. coli* as the most commonly occurring organism. This is also a common organism in non-diabetics [6], Female gender had a higher predisposition to the occurrence of UTI. Our study showed more infection in age 40 and above which could explain hormonal and muscular changes observed with the progressive age particularly in females [9]. HbA1C levels were not found to be significantly associated with UTI status but those with positive cultures had Hba1c ≥7% and were poorly controlled.

Among patients with type 2 diabetes, both symptomatic and asymptomatic urinary tract infections occur more frequently as compared to the general population without type 2 diabetes disease [10]. Corresponding to the prevalence of asymptomatic UTI in our study (8.1%), previous studies reported the prevalence of asymptomatic UTI in diabetic population between 8 and 26% [11–13]. Dominant presence of E.coli as most common organism in our study consistent to what recently reported earlier in similar population [6] with increasing trend of antibiotic resistance in the region for urinary tract infections pose a greater than expected threat to diabetic population [14].

A study based on administrative data of the United States population revealed a higher incidence of UTI in female versus male gender (12.9% vs. 3.9%) during a year [15]. In addition to this, the tendency of female gender for UTI is also reported in previously published studies with a geographical population like ours [16]. The higher occurrence is related more to the anatomy of the female urinary system short urethra and bacterial colonization in the perianal area and less associated with physiological changes in the body due to diabetes [17].

Distribution of positive urine culture in type 2 diabetes with respect to gender though, has diverse reporting, with somewhat underreporting for the male gender. The prevalence of UTI in female gender was reported in previous literature [18] which also showed in our study results.

Majority of the patient had HbA1c levels 7 and above, were not related to negative or positive UTI status of the patients, having an insignificant association. This strengthens the fact that the severity of derangement in HbA1c does not necessarily affect the biological flora or has any role in UTI susceptibility as reported in a meta-analysis of 22 studies [19].

The causal relationship of UTI among diabetic female and age > 40 years was not possible due to limitation of cross-sectional study design. The study also lacks the evaluation of confounding factors like socioeconomic status and education. However, the study results presented with a healthy sample size covering the multiple sites across Pakistan.

## Conclusion

Diabetic female patients are identified to be at high risk of suffering from asymptomatic UTI and age more than 40 years can significantly play an important role. There was more risk of infection in females with Hba1c 7 and above. *Escherichia coli* was the most common causative organism among patients with diabetes living in this geographical area.

## Data Availability

The datasets used and/or analysed during the current study will be available from the corresponding author upon request.

## Abbreviations

T2DM: Type 2 Diabetes mellitus
UTI: Urinary tract infections
HBA1c: Glycated hemoglobin
ALT: Alanine aminotransferase
ALP: Alkaline phosphatase
AST: Aspartate aminotransferase
eGFR: Estimated glomerular filtration rate
E.coli: *Escherichia coli*
